# Department of Surgery

**Published:** 1899-02

**Authors:** L. A. Merillat

**Affiliations:** Secretary of M’killip Veterinary College, Chicago


### DEPARTMENT OF SURGERY.

By L. A. Merillat, V.S.,
SECRETARY OF M'KILLIP VETERINARY COLLEGE, CHICAGO.

WITH THE COLLABORATION OF

W. E. A. Wyman, M.D.V., V.S.,
MILWAUKEE, WIS.

AND OTHERS.

### Antiseptics.

Aseptic treatment is not always possible in veterinary practice, yet the veterinarian must adopt and follow the rules which govern aseptic surgery in all his operations to gain the best possible results. In doing so, however, there need be no surprise if in some instances the results do not correspond to the precautions taken.

To sterilize surroundings, patient, assistants, grooms, bedding and casting appliances, besides the hands, instruments, and dressings which would be necessary to obtain "aseptic results" in all cases, is a task not expected of the veterinarian, and one which can seldom be accomplished. But this must not be made an excuse for the careless, unclean surgery so common in veterinary operations. It is a serious reflection upon the veterinary practitioner that a large percentage of operations are performed without regard to asepsis or even ordinary cleanliness. How often do we simply dip the knife into an antiseptic solution, sponge the wound or seat of operation carelessly, perform the operation with unclean hands, sew up the incision with unprepared sutures, or dress the part with at least doubtful dressings, and then wonder why the procedure was not a success?

It is hardly necessary to mention that all septic processes are caused by organisms introduced (with few exceptions) from without by the hands, instruments, dressings, and lotions, or by floating particles. Granting this to be true, it is evident that much can be done to prevent their invasion even under the most adverse circumstances. Extensive inflammation, swelling, œdema, fever, pus, etc., which detract from the value of so many veterinary operations, are in most cases avoidable, and hence due to negligence of the surgeon.

The following four rules, if carefully executed, will give suitable results in most operations:

I. Purify the seat of operation.

II. Purify the hands and instruments.

III. Destroy the organism which may fall upon the wound during the operation.

IV. Apply an antiseptic dressing which will prevent invasion during the healing process.

I. *To purify the seat of operation.* While it is readily seen that none of the above steps can be omitted or even slighted without rendering the others useless, none is more important than the sterilization of the surgical field as well as a liberal surface around it.

To accomplish this the following method has proved effectual:

1. Clip the hair closely (shaving is difficult and unnecessary).

2. Scrub with soap and water at 125° F. This is done thoroughly with a stiff hand-brush.

3. Dry with a sterilized cloth and apply ethyl-alcohol with considerable friction.

4. Wash for a few moments with a mercuric chloride solution 1 : 500.

5. The foregoing steps may be effected twenty-four hours before the operation and the part covered with a bandage soaked in a saturated solution of sodium chloride, or the operation may begin immediately after the fourth step.

II. *To purify the hands and instruments.* The hands are washed in the usual manner and then dipped and rinsed in a mercuric chloride solution, 1 : 500. The latter must be repeated continually during the operation, especially when the hands touch any part not sterilized.

Instruments are boiled in a 1 per cent. solution of sodium carbonate (washing soda) and kept in a shallow tray containing a 3 per cent. solution of carbolic acid. This latter precaution would seem cowardly to the human surgeon or at least reflect seriously upon the assistant who is charged with sterilizing the instruments, but the veterinarian is ever mindful of the contaminated atmosphere in which he operates. When any one instrument is used for a considerable time it is repeatedly dipped into the same solution and always replaced there when not in use.

III. *To destroy the organisms which may fall upon the wound during the operation.* For this purpose there is nothing equal to a mercuric chloride solution, 1 : 1000, or even 1 : 2000. A large quantity of such a solution must always be at hand, and the wound must be repeatedly irrigated during the entire procedure, from the very time the first incision is made until the part is perfectly covered with an impervious antiseptic dressing.

The solution, unless made with distilled water, is prepared only for immediate use. There should be a small vessel (strength, 1 : 500) for the surgeon to dip the hands, and a large one (strength, 1 : 1000, 1 : 1500, or 1 : 2000) for the assistant who attends to the irrigation and bailing. The assistant uses an extra vessel, a porcelain bowl, so that the blood-sponges need never be carried into the large receptacle. Blood, as well as the constituents of tap-water, greatly impair the potency of mercurial solutions.

IV. *To apply an antiseptic dressing which will prevent invasion of septic organisms during the healing process.* The application of suitable dressings is a difficult problem in many veterinary operations. So long as the operation is confined to the lower portion of the extremities, bandages can be applied to good advantage; but when any other part of the body is subjected to surgical treatment the surgeon is at once at sea for an exclusive and appropriate method of excluding external influences. The tyro will attempt but once to bandage the thigh, stifle, elbow, shoulder, flank, or even the hock. The latter may sometimes be successfully bandaged, but in most cases the patient will do more damage in fighting the bandage than if the wound had been left exposed. I know of no method of successfully applying bandages to the above-mentioned regions. One may make exceptions of the poll, withers, and umbilical region, but even these are kept covered with difficulty. It is, therefore, evident that the veterinary surgeon must look about for a means of excluding organisms without the use of bandages. But what?

Let us take, for an example, a draught-horse with a large shoulder tumor on the mastoido-humeralis. The animal is prepared in strict conformance to the above rules, and the tumor is carefully excised. As soon as the dressing is to be applied the surgeon is confronted with extensive hemorrhage. No form of external dressing will arrest it, so the cavity is filled tightly with sterilized, or, what is still better, antiseptic packing (oakum or cotton) and the incision sewed up. If left in this condition the packing is a putrid mass in less than thirty-six hours. Knowing the cause of this putrefaction so well, is it not possible to apply a remedy? Collodion will not adhere to a wet surface, and when applied to a dry surface it is nothing less than a nuisance when dressing the wound becomes necessary. Dry antiseptics (boric acid, quinine, antifebrine, iodoform, etc.) may be applied and covered over with a thick coating of carbolized vaseline; but this is not exclusive, as the vaseline will soon melt and carry off the whole dressing. Plasters on thin gauze made with resin, oils, and some antiseptic are probably the most suitable, but have much the same disadvantages as collodion in being difficult to handle, to remove, and to reapply, and being impervious they prevent the discharge of the exudate of reparative inflammation.

For a wound that requires repeated dressing the following plaster has many advantages. It does not desiccate, and is therefore easily removed:

| | |
|---|---|
| ℞.—Oleum ricini . . . . . . . . | 8 parts. |
| Resin . . . . . . . . . | 14 " |
| Acid. carbol. . . . . . . . . | ½ part. |

Mix over a slow fire and spread over cloth, oiled silk, or paper. Apply warm.

For simple incisions where primary union is expected linseed oil is substituted for castor oil. This mixture will desiccate and form a hard coating, which is removed with more difficulty than the first. Either alcohol or oil of turpentine are the best solvents to remove them.

These by no means possess the desired quality, and there remains to be discovered some impervious substance that will adhere to the wet or bloody skin and at the same time be easily removed.

On the extremities gauze, oakum, muslin, and flannel bandages are used with universal success. All such dressings, when soaked in a mercuric chloride solution, 1 : 500, will prevent septic invasion indefinitely. The gauze, oakum, or cotton is first covered with a number of layers of muslin, and this in turn with a red flannel bandage. Red flannel is selected because blood from the wound and finger-marks do not give the dressing an unsightly appearance, as is always the case when only white is used.

*Heat* at 212° F. is the best of all antiseptics. It is used in veterinary surgery in the form of boiling water to sterilize instruments, bandages, and other forms of dressings and appliances to be used in operations.

*Mercuric chloride.* It requires only a few experiments to illustrate the value of this drug to the veterinary surgeon. It has been frequently superseded by other salts of mercury, zinc, silver, and various coal-tar derivatives, but in every case has regained its high place in the list of antiseptics. It has been condemned for preventing primary union and its corrosive effect upon instruments.

The former effect is due to the use of a too concentrated solution; as for the latter, it need never be used upon them.

For disinfecting the seat of operation, washing the hands before and during the operation, and for soaking bandages, it should be used in a 1 : 500 aqueous solution. For irrigating the wound during the operation and for disinfecting a recent accidental wound that has been exposed, 1 : 1000 to 1 : 2000. As a general antiseptic in the treatment of granulating and suppurating wounds, 1 : 1000.

It should never be prescribed with ordinary tap- or well-water except for immediate use, as it is readily thrown down as insoluble chlorides. With blood it forms insoluble albuminates and immediately loses its potency.

*Carbolic acid* is one of the oldest and most effectual antiseptics. It is utilized in a 2 to 3 per cent. aqueous solution for instruments during operation and in the treatment of foul-smelling and suppurating wounds generally. Carbolic acid was formerly used as a spray during operation, but this precaution has never been popular with the veterinarian, and is considered unnecessary by the human surgeon.

*Creolin* deserves no place in veterinary surgery, except possibly in obstetrical operations. Repeated attempts to perform aseptic operations with it have always resulted in disappointment. Its cheaper substitutes—germol, chloronaptholeum, etc.—are useful in disinfecting large foot-baths, but their only redeeming feature is their small cost.

*Zinc chloride* in the form of Burnett's disinfectant fluid is said to prevent septic processes in exposed wounds for several days. It has, however, never been popular.

*Dry antiseptics* are useful as well as essential. They have the advantage of remaining where they are placed longer than liquids. The favorite ones are acetanilid, quinine, boric acid, iodoform, thioform, and tannic acid. Their relative worth is difficult to ascertain. Boric acid ranks high and has the advantage of being non-irritating. Squibb's compound alum-powder, although only recently introduced, is leaving a good impression wherever used, and will doubtless win a place in the veterinary pharmacy.

The following table contains the antiseptics commonly used in veterinary practice, named in the order of their strength. The figures are the minimum dose capable of preventing putrefaction in one litre of neutral beef bouillon:

| | | | | | |
|---|---|---|---|---|---|
| 1. | Mercuric iodide | .025 grams. | | .3875 grains. | |
| 2. | Mercuric chloride | .07 | " | 1.085 | " |
| 3. | Argent. nitras | .08 | " | 1.24 | " |
| 4. | Chlorine | .25 | " | 3.875 | " |
| 5. | Iodine | .25 | " | 3.875 | " |
| 6. | Iodoform | .7 | " | 10.85 | " |
| 7. | Cupric sulphate | .9 | " | 13.95 | " |
| 8. | Acidum salicylicum | 1. | " | 15.5 | " |
| 9. | Zinci chlor. | 1.9 | " | 29.45 | " |
| 10. | Acidum carbol. | 3.2 | " | 49.6 | " |
| 11. | Potassæ permangan. | 3.5 | " | 54.25 | " |
| 12. | Acidum boricum | 7.5 | " | 1.93 drachms. | |
| 13. | Borax | 70. | " | 2.25 ounces. | |
| 14. | Ethyl-alcohol | 95. | " | 3. | " |
| 15. | Sodii chlor. | 165. | " | 5.325 | " |
| 16. | Glycerin | 225. | " | 7.5 | " |
| 17. | Sod. hyposulphis | 275. | " | 8.8 | " |

This scale refers only to organic decomposition—putrefaction—and will not apply to all or any one of the pathogenic bacteria. Particular organisms may readily succumb to certain drugs which in turn offer little resistance to others; and, again, the time given them to act, the number and vitality of the organisms present, the tissues in which they are located, and the medium in which they are dissolved must all be taken into account in the study of antiseptics. In fact, their action is altered by so many conditions that no scale, however carefully prepared through experimentation, fully defines their relative worth under all circumstances.

L. A. Merillat.

---

### Clinical Diagnosis of Stringhalt.

From a clinical standpoint stringhalt may be divided into two classes—true and false stringhalt.

Stringhalt consists of a spasmodic, excessive flexion of the articulations of the swinging leg, unilateral or bilateral, more or less permanent, depending on various visible and obscure states.

True stringhalt subdivides itself into two main forms. In the one there is a distinct forward-upward movement, the metatarsal bones being elevated until in serious cases the fetlock is raised as high as the hock, or, in other words, the metatarsus practically lies in a horizontal plane with the linea alba. The other type is characterized by a backward and upward movement of the hock and foot. At the same time modifications of the above, as either the forward-upward or backward-upward movement with more or less abduction, etc., are observed.

These abnormalities, either intermittent or permanent, are best detected at a walk, or when the animal is rotated or induced to step from one side to the other. Generally speaking, it is less pronounced at a trot, and barely visible at a gallop.

True stringhalt, being a symptom accompanying a number of obscure causes, is necessarily of marked interest from a clinical standpoint, especially as to prognosis and treatment. Apropos, stringhalt offers a fair example to encourage every surgeon to closely study physiology, pathology, and clinical diagnosis, and apply the knowledge thus gained to surgery; the longer and the more we devote ourselves to the study of drugs, the more one realizes the fact that the practitioner who is knife-shy succeeds only partly.

True stringhalt of the forward-upward type is either very much improved or completely cured by peroneal tenotomy combined with cutting of the tibial fascia, while the backward-upward form is frequently not beneficially influenced by such surgical interference.

The Italian operation by Bassi, if I am not mistaken, consists of the cutting of the internal patellar ligament, an operation offering more difficulties than claimed by its originator. Bassi claims that true stringhalt is due to retraction of said ligament, while Dieckerhoff looks upon retraction of the tibial fascia as a primary cause of stringhalt. Other authors, too many to enumerate, give as causes an interstitial neuritis of the ischiatic nerve, diseases of various joints, mainly the coxo-femoral articulation, etc. All these, excepting Bassi's and Dieckerhoff's theory, are lacking in proof.

The two most plausible explanations for stringhalt are: True stringhalt follows a shortening or retraction of ligaments, fascia, or tendons, interfering more or less with normal flexion of the lower articulations, as the result of which the flexor muscles are called into active play, causing excessive flexion, and thus elevation of the joints.

False stringhalt being symptomatic, and a reflex act, either acute or chronic, is noticeable in diseased conditions of the flexion surface of the hock or fetlock, as, for instance, scratches, toe- and quarter-cracks, injuries to the coronary region, spavin, ringbone, grease, chronic cellulitis, etc.

To summarize: From a clinical point of view stringhalt should be divided into:

1. True or idiopathic stringhalt. Cause or causes obscure, most likely due to retraction of ligaments, tendons, and fascia.

2. False or symptomatic stringhalt.

(*a*) Temporary or acute.
Scratches, injuries to coronet, cracked hoof.
Injuries to flexion surface of hock and fetlock.

(*b*) Permanent or chronic.
Spavin, curb, ringbone, chronic changes in stifle and hock-joints.
Chronic cellulitis, grease, chronic disease of the frog, keloids in flexion surface of hock and fetlock.

W. E. A. Wyman, M.D.V., V.S.

---

### Surgical Items.

Carbolic acid must be avoided as a surgical dressing for dogs. Even when used sparingly it is often attended with organic disturbances, if not death.—Ed.

The amount of absorbent dressing over a wound must be gauged by the amount of effusion of blood and serum likely to occur. It is better to err on the side of extra-copiousness. A scanty dressing necessitates frequent change, since saturation of the dressing defeats purposes of asepsis.—*Int. Journ. Surg.*

If a nerve be immediately sutured, or if a gap caused by resection be immediately bridged with some suitable material, and the work done aseptically and properly, but slight degenerative changes will take place either in the distal or proximal portion of a nerve; and the nerve's function will in a large majority of cases be quickly re-established.—A. V. L., *Int. Journ. Surg.*

Reunion of the severed nerves following the various neurectomy operations in the horse is ordinarily the result of tardy healing of the incision. When the incision heals by immediate union the distal portion promptly degenerates, and a post-mortem several months later scarcely reveals its existence.—Ed.

Soapy water is a poor solvent for blood. Bloody hands should be first washed in pure water, then with soap.—*Ibid.*

Cutting instruments should possess a razor-like sharpness. A grindstone and an oilstone are almost indispensable equipments to the veterinary operating-room, and the veterinarian can do no better than to spend some of his leisure hours at sharpening instruments. Dull knives cause many a bungling, prolonged, and unsuccessful operation.—*Ibid.*

One of the best astringents to control granulations is made of 30 grammes (1 ounce) each of zinci sulphas, plumbi acetas, and cupri sulphas in 960 grammes (1 quart) of water.—*Ibid.*

